# Supplementary material for: Genome-Wide Identification and Characterization of Long Non-Coding RNAs in Roots of Rice Seedlings under Nitrogen Deficiency
Source: Plants (Basel). 2023 Nov 30;12(23):4047. doi: 10.3390/plants12234047 (PMC10708291; doi:10.3390/plants12234047)
Supplement: Supplementary file 1 [file plants-12-04047-s001.zip › Table S10.pdf]

Table S10. List of the primers in this study.

Primer Name	Sequences(5'-3')	Notes
**Primers for qRT-PCR**		
eEF-1 qRT/F	GCACGCTCTTCTTGCTTTC	Os03g0178000
eEF-1 qRT/R	AGGGAATCTTGTCAGGGTTG	
MSTRG24486.1 qRT/F	ACTCCTATGTGGCTACTG	
MSTRG24486.1 qRT/R	GCAAGAATGATACTACGC	
MSTRG24177.3 qRT/F	CGGACGATCAATCCTTCCCG	
MSTRG24177.3 qRT/R	TAGTCCCGGATTGGTAGCGA	
MSTRG 24320.6 qRT/F	TCGCTCAAAGCTGCTCTCTC	
MSTRG 24320.6 qRT/R	ATTACCCCCTGCCTTACCCT	
MSTRG 1614.2 qRT/F	TGGAAACACCGAGCACTCAA	
MSTRG 1614.2 qRT/R	TCCAGTGGGATCTTCAACGC	
MSTRG 12144.19 qRT/F	TCCATCGCTGCCATGAACTC	
MSTRG 12144.19 qRT/R	TCACATTGCTAGCGTTCACCT	
MSTRG 22856.2 qRT/F	CAAACCCTAGGTGGTGGTCC	
MSTRG 22856.2 qRT/R	CCATAGTTAGCCGAGCGGAG	
MSTRG 26592.1 qRT/F	TGCTATCTGATGAATGATGTCGT	
MSTRG 26592.1 qRT/R	AGCATCCATATTGTTTACACTTGTT	
MSTRG 10516.1 qRT/F	TGAAGAGAAGCCCTTGTCGG	
MSTRG 10516.1 qRT/R	GCCAGGTAGGCCAACAACTA	
LOC4330076 qRT/F	GGGTGGTTGAAAGGGTAGGG	Os02g0635000, bifunctional nitrilase/nitrile hydratase NIT4-like
LOC4330076 qRT/R	GCTGTCCTGAGAAGTGGCAT	
LOC107278693 qRT/F	GGTCGCAGAACAAAGCTGAC	Os04g0339500 germacrene D synthase; alpha-humulene synthase
LOC107278693 qRT/R	CACATCACGGTGGTCAATGC	
LOC4344505 qRT/F	TTCCACCCGTCCAACAGC	Os08g0113900, uncharacterized protein
LOC4344505 qRT/R	TGTCGATTACCACCATGGACA	
LOC107277478 qRT/F	CGAGGCGGTTCGGGATG	Os01g0547600, high-affinity nitrate transporter 2.4
LOC107277478 qRT/R	TTTGCAGACGAAGGGAACGA	
LOC4345353 qRT/F	ACGGCTTCATCGTACCACAA	Os08g0352100 uncharacterized protein
LOC4345353 qRT/R	GTCGTAGTAGCCAGCACCTC	
LOC4348926 qRT/F	AGCTCTACGCCAAGCAGAAG	Os10g0481500, flotillin-like protein 1，Nodulin-like protein 1
LOC4348926 qRT/R	TTGCTCCACACGCTGATCTT	
LOC107276221 qRT/F	GTGAACTCCGAGATCGACCG	Os11g0671200, uncharacterized protein
LOC107276221 qRT/R	CATCTTGTCCTCGCCGTCAT	
Os09g0263933 qRT/F	GAGGTCCTGAAGAATCCGGC	LOC_Os09g08920.1, flavonoid 3-monooxygenase
Os09g0263933 qRT/R	GACATCCGGGGTATCAGCAG	

Os09g08890.1 qRT/F	TGACTTTGCACGACAAAGCG	LOC_Os09g08890.1
Os09g08890.1 qRT/R	TCCGGGGAGAATTCGCAAAA	
Os09g08900.1 qRT/F	CACCGGAGGTTGCTTGTTTG	LOC_Os09g08900.1
Os09g08900.1 qRT/R	ATAACTTGGGCAAGGGTCGG	
Os09g088910.1 qRT/F	GGTCGAGTGGTCTCAGTTGG	LOC_Os09g08910.1
Os09g088910.1 qRT/R	GGCCTAACATGGCCTTTCCT	
Os09g088930.1 qRT/F	CACAATCCGCACAGCAAACA	LOC_Os09g08930.1
Os09g088930.1 qRT/R	CCCGTTGCAACCTCCTTAGT	
**Primers for vector construction**		
lncRNA24320.6 Pro F	GGGGTACCTGACGGATCGGAGGGACT	For cloning and construction of lncRNA24320.6 promoter
lncRNA24320.6 Pro R	CATGCCATGGATGGAAGGCGGGATGAAAC	
lncRNA24320.6 OE-F *Hin*d III	GCTAAGCTTCCCCGCATTTCGCTCAAAGC	For cloning the full length of lncRNA24320.6
lncRNA24320.6 OE-R *Bam*HI	GCTGGATCCTAAAAGCTTTCAAACTCAGT	
Hpt-t/F	GATGTTGGCGACCTCGTATT	
Hpt-t/R	TCGTTATGTTTATCGGCACTTT	
