# Supplementary figures and images for: Genome-Wide Identification and Characterization of Long Non-Coding RNAs in Roots of Rice Seedlings under Nitrogen Deficiency
Source: Plants (Basel). 2023 Nov 30;12(23):4047. doi: 10.3390/plants12234047 (PMC10708291; doi:10.3390/plants12234047)

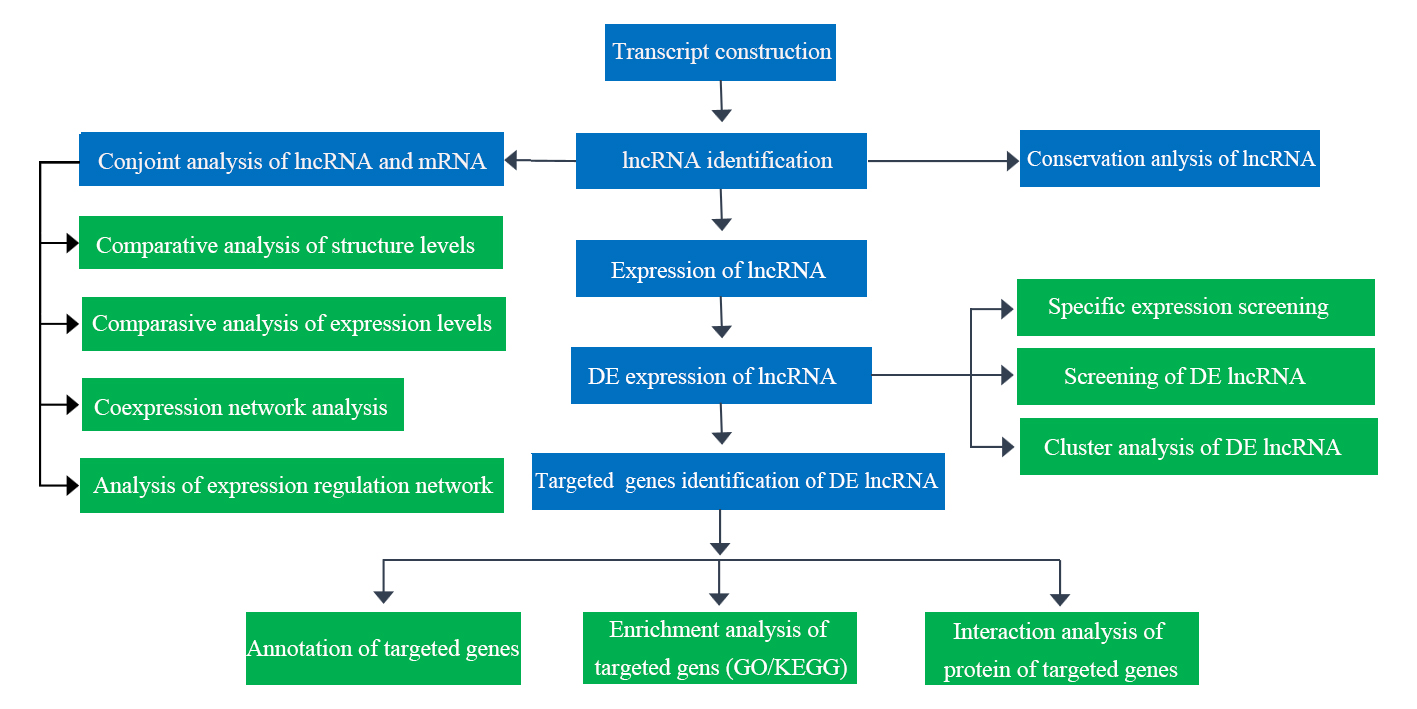

Supplement: Supplementary file 1 [file plants-12-04047-s001.zip › Fig S1.jpg]

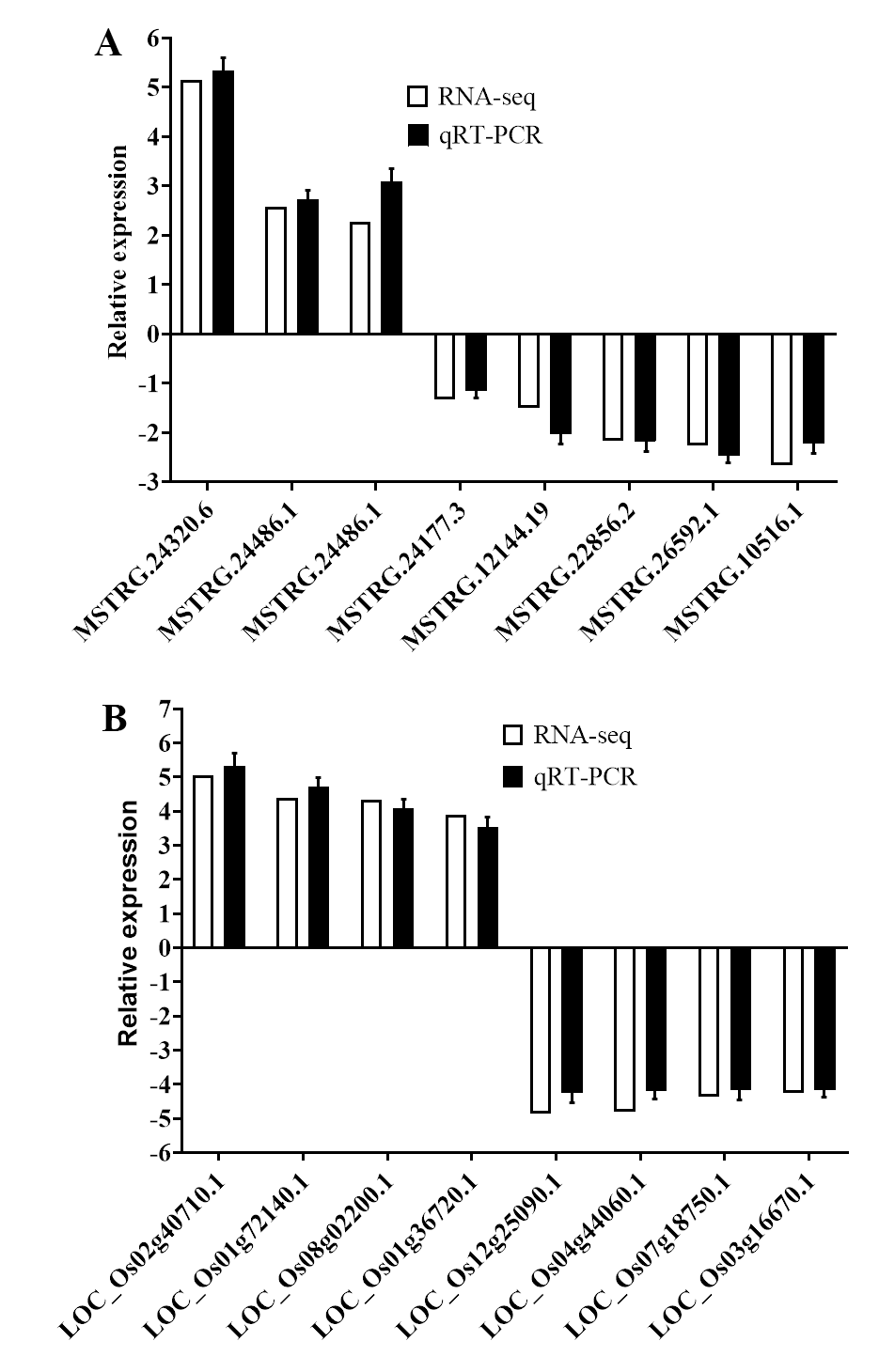

Supplement: Supplementary file 1 [file plants-12-04047-s001.zip › Fig S2.tif]

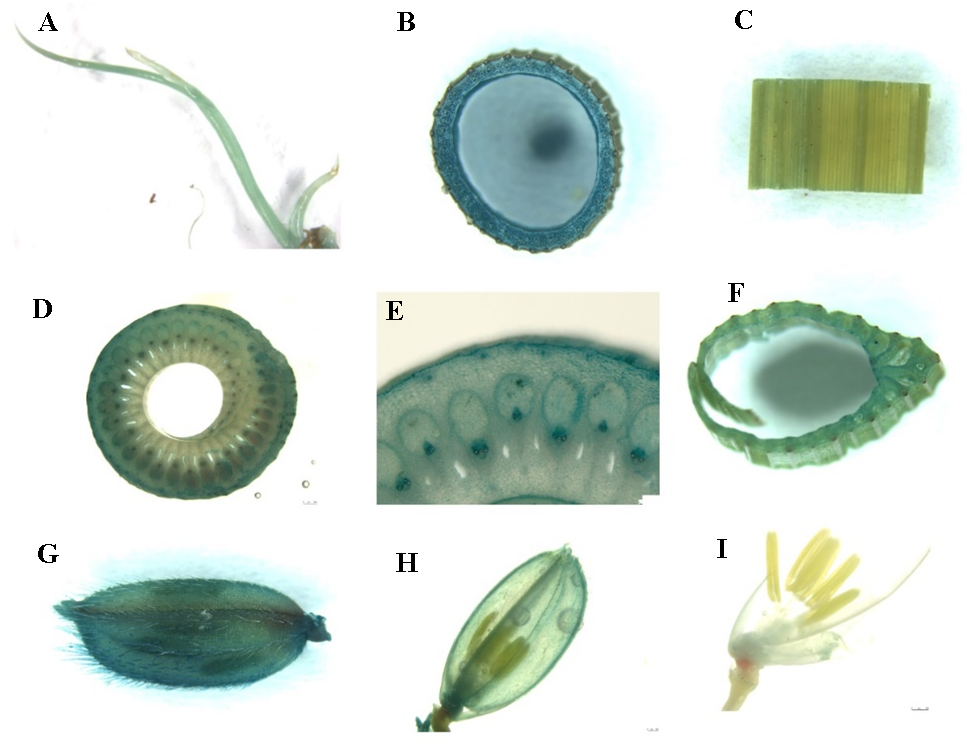

Supplement: Supplementary file 1 [file plants-12-04047-s001.zip › Fig S3.tif]

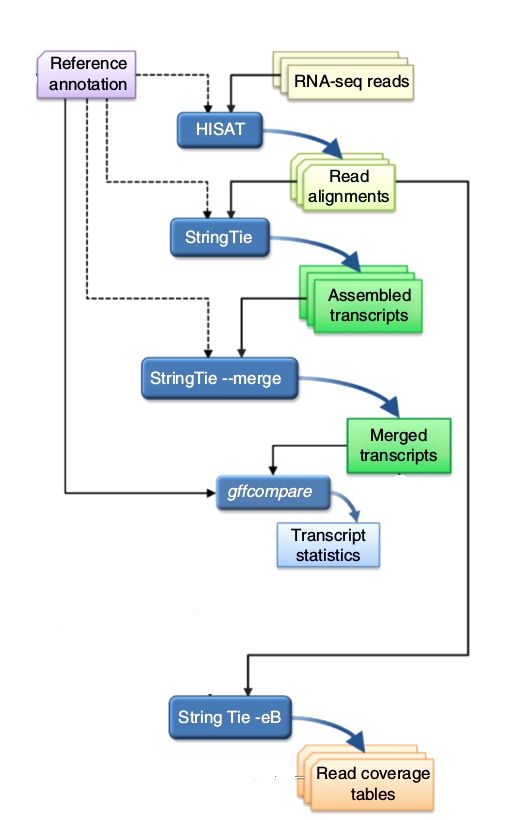

Supplement: Supplementary file 1 [file plants-12-04047-s001.zip › Fig S4.png]
